# Measles Vaccination in Presence of Measles Antibody May Enhance Child Survival

**DOI:** 10.3389/fped.2020.00020

**Published:** 2020-02-07

**Authors:** Christine S. Benn, Cesário L. Martins, Andreas Andersen, Ane B. Fisker, Hilton C. Whittle, Peter Aaby

**Affiliations:** ^1^Bandim Health Project, Indepth Network, Bissau, Guinea-Bissau; ^2^Research Centre for Vitamins and Vaccines, Bandim Health Project, Statens Serum Institut, Copenhagen, Denmark; ^3^Odense Patient Data Explorative Network, Odense University Hospital, Institute of Clinical Research, University of Southern Denmark, Odense, Denmark; ^4^Department of Infectious and Tropical Diseases, The London School of Hygiene and Tropical Medicine, London, United Kingdom

**Keywords:** measles, vaccine, child mortality in Africa, maternal antibodies (matab), heterologous (non-specific) effects of vaccines

## Abstract

**Background:** In trials of early two-dose measles vaccination (MV), with the first dose being given before 9 months of age, vaccination in the presence of maternal antibody reduced mortality 2- to 3-fold compared with MV in the presence of no measles antibody. We tested this finding in two historical studies in which the children had received one dose of MV.

**Methods:** We used data from a surveillance study of seroconversion after standard-titer MV (Schwarz strain) (Study 1) and a trial of early medium-titer MV (Edmonston-Zagreb strain) in which a pre-vaccination blood sample had been collected (Study 2). Both studies had control children, who were enrolled under similar conditions, but did not receive effective MV. Study 1 was a natural experiment where all children measles vaccinated during 1 month did not seroconvert and had therefore received an ineffective vaccine. In Study 2, the controls were randomized to an inactivated polio vaccine (IPV). We compared mortality for children with undetectable levels of measles antibody (<31.25 mIU) at baseline with children with detectable levels (≥31.25 mIU).

**Results:** In both studies, children who were measles vaccinated in the presence of measles antibody had lower mortality compared with children who were measles vaccinated in presence of no measles antibody, the combined mortality rate ratio (MRR) being 0.51 (0.27–0.96). In the control groups, a detectable level of measles antibody vs. an undetectable level was not associated with lower mortality, the MRR being 1.40 (0.31–6.38).

**Conclusion:** The results supported previous findings: measles vaccination in the presence of measles antibody had beneficial effects on child survival. Since maternal antibody levels are declining, it may be time to consider giving MV earlier and/or to provide MV to adolescent girls to boost antibody levels.

## Introduction

Studies from low-income countries indicate that measles vaccination (MV) has beneficial effects on child survival when given earlier rather than later (1–7). This contradicts assumptions underlying the global policy (8–10): Clinical vaccine efficacy (VE) against measles infection improves when MV is administered after maternal antibody has waned. High-income countries with low incidence of measles infection therefore provide MV at 12–18 months of age. However, low-income countries administer MV at 9 months of age since the incidence of measles is high among infants (8). WHO recommends that once measles is under control, the age of vaccination should also be raised to 12 months of age, as happened in Latin America in 1996 when measles was eliminated (10).

However, the effect of MV on child survival may not merely be due to protection against measles infection; MV seems to protect against death from other infections (1–4, 11). One potential explanation for this effect could be that these beneficial non-specific effects (NSEs) of MV are more pronounced when vaccination is given early in the presence of measles antibody. This hypothesis was supported in the *post-hoc* analysis of two randomized trials of an early two-dose vaccination schedule, in which we had collected pre-vaccination blood samples (3, 12). In both study cohorts, overall mortality was reduced between 4 months and 5 years of age if early MV was given in the presence of maternal measles antibodies vs. no maternal measles antibody (13). The studies involved early two-dose MV schedules. In this paper, we test the hypothesis by analyzing two historical datasets in which only a single dose of MV was used.

## Methods

We used two studies conducted in the urban study area of the Bandim Health Project (BHP), Guinea-Bissau, in which a pre-vaccination blood sample had been collected at baseline, prior to MV (6, 13). Both studies had a group of control children, who had pre-vaccination antibody levels measured at baseline but did not receive an active MV.

In the mid-1980s when these studies were conducted, measles infection was widespread. Hence, there is no way of knowing whether measles antibody measured in infancy are maternal or due to clinical or sub-clinical measles infection. Since maternal antibodies are declining with age of the child, it is likely that lower levels are maternal in origin whereas higher levels are due to measles exposure or undocumented measles vaccination. After measles infection or measles vaccination we have usually found that the children had antibody levels of 500 m IU/ml or above, whereas most young infants aged 4–5 months with detectable measles antibody and no history of measles infection have samples in the 31.25–250 m IU/ml range (14, 15). Hence, among children with measurable antibodies, we have separated those with low level in the range of 31.25–250 mIU/ml, probably of maternal origin, and those with higher levels probably due to previous measles exposure. We compared those with undetectable levels with those with low levels and with those with any level of antibody.

### Study 1. Surveillance of Routine Schwarz Measles Vaccine

Due to concern over many cases of measles infection among vaccinated children, we monitored measles vaccinations in the Bandim 1 study area between May 1984 and March 1985 (6); 227 children had a pre-vaccination and post-vaccination sample collected on filter-paper. Due to problems in the laboratory in Copenhagen, samples were only analyzed 2 years later and it turned out that during a 3-weeks period, vaccinated children had not seroconverted indicating problems with the vaccine. The 166 children who received an effective MV are group MV1 (Table 1). The 61 children who received the inactive MV are a natural-experiment control group vaccinated with a “placebo” (group C1, Table 1); though not randomized, it was a “blind” control group. The control group had a slightly higher mortality level than the MV1 group, presumably because children did not receive an effective MV before the end of follow-up in the study. At the time, the official policy was MV from 9 months of age. The median age of vaccination was 361 days. Follow-up was to 3 years of age. Once the error was detected the “placebo” group was offered a new MV.

**Table 1:** Mortality in relation to the presence of measles antibody at the time of measles vaccination or control vaccination.

**Measles antibody concentration in pre-vaccination sample (mIU/ml)**	**Deaths/number**			
	**Study 1, MV1**	**Study 1, C1** **(inactive MV)**	**Study 2, MV2**	**Study 2, C2** **(IPV)**
<31.25	8/100	2/36	14/66	12/72
31.25	1/29	4/14	4/16	1/18
62.50	0/10	1/6	2/35	5/31
125			6/39	4/32
250			0/18	4/17
500	0/4		3/10	0/4
1,000	0/4		0/4	0/5
2,000	0/19	0/5	0/1	0/3
4,000			0/1	0/1
8,000			0/2	0/1
16,000			0/1	0/1
All	9/166	7/61	29/193	26/185
MRR (31.25–64,000 mIU/ml vs. undetectable level)	0.19 (0.02–1.48)	3.60 (0.76–17.11)	0.56 (0.29–1.08)	0.74 (0.36–1.52)

*MV1, Children received an effective MV; C1, Children received an inactive MV; MV2, Children received EZ at 4–8 months and possibly IPV at 9 months; C2, Children received IPV at 4–8 months of age and possibly standard MV at 9 months of age*.

### Study 2. Trial of Medium-Titer Edmonston-Zagreb (EZ) Measles Vaccine

From February 1985, children born between August 1984 and September 1985 and registered in Bandim 1 were enrolled in a trial of medium-titer EZ MV (14, 16). The children were randomized to EZ or inactivated polio vaccine (IPV) from 4 months of age. At 9 months, they were invited back; the EZ group received IPV and the control group received standard titer Schwarz MV. Thus, group MV2 received EZ at 4–8 months of age and possibly IPV at 9 months; and group C2, the control group, received IPV at 4–8 months of age and possibly standard MV at 9 months of age (Table 1). Pre-vaccination samples were collected from all children at the time of the first vaccination at 4–5 months of age. Based on information about the cause of death obtained from the parents, two deaths were due to accident and were excluded in the analysis. We collected information on the main symptoms at the time of death to be able to exclude non-infectious deaths.

The median age of vaccination was 157 days. Follow-up was to 5 years of age (14). There was no over-lap between the children in Study 1 and Study 2.

#### Measles Antibody Assays

In the previous studies of MV and maternal antibodies the antibody level was measured using a hemagglutination inhibition (HAI) test (13, 16). The minimum detectable level of measles antibody with this assay was 31.25 mIU. In study 1, samples had only been analyzed with a measles IgG enzyme-linked immunosorbent assay (ELISA) (6). In study 2, both HAI and ELISA assays had been used (17) and we therefore used these data to assess the correspondence between the two assays. A ROC analysis identified the ELISA cut-offs that best corresponded to the undetectable level of HAI antibodies and the positive HAI measurements. The ELISA cut-offs were then used to determine the level of undetectable/low measles antibody in study 1.

#### Statistical Analyses

Using a Cox proportional hazards model with age as underlying time, we compared the mortality rate ratios (MRRs) of groups having different concentrations of pre-vaccination antibody to assess the importance of antibody at time of MV for child survival.

## Results

The distribution of pre-vaccination antibody levels and deaths for all children in the two studies is shown in Table 1. When we compared the mortality of MV vaccinated children with any baseline level of measles antibodies with those with undetectable baseline levels, the mortality rate ratio (MRR) was 0.19 (0.02–1.48) and 0.56 (0.29–1.08) in the two studies, for a combined estimate of 0.51 (0.27–0.96). Compared with children with undetectable levels, children with low detectable levels (31.25–250 mIU), that were likely to have been maternally derived, had lower mortality following MV in both studies, the combined MRR being 0.48 (0.24–0.95) (Table 2).

**Table 2:** Mortality according to level of measles antibody at time of vaccination with measles vaccine or control vaccine.

	**Mortality rate per 1,000 person-years (Deaths/person-years) [N]**					
**Study reference and type of measles vaccine (MV)**	**Measles vaccination**			**Control vaccine#**		
	**Had measles antibody (31.25–250 mIU/ml)**	**No detectable measles antibody**	**Mortality rate ratio (95% CI)** **(detectable/undetectable)**	**Had measles antibody (31.25–250 mIU/ml)**	**No detectable measles antibody**	**Mortality rate ratio (95% CI) (detectable/undetectable)**
Study 1 (6), Schwarz MV	26 (1/39)	80 (8/100)	0.32 (0.04–2.48)	250 (5/20)	56 (2/36)	4.50 (0.96–21.1)
Study 2 (13), EZ MV	111 (12/108)	212 (14/66)	0.52 (0.26–1.06)	143 (14/98)	169 (12/71)	0.85 (0.42–1.72)
Combined			0.48 (0.24–0.95)			1.69 (0.34–8.42)

*Study 1: controls received ineffective MV; Study 2: controls received inactivated polio vaccine*.

In the control groups that did not get an active MV at enrolment, there was no benefit from having detectable baseline levels of measles antibody compared with undetectable baseline levels, the combined MRR being 1.40 (0.31–6.38). Hence, the effect on child survival of measles antibody tended to differ depending on whether the children received MV or a “control” vaccine (ineffective MV, IPV) (test of same effect of having detectable baseline levels in those MV vaccinated at enrolment and those not MV vaccinated at enrolment, *p* = 0.23).

## Discussion

This analysis of historical data confirmed the hypothesis that MV in the presence of measles antibodies was associated with lower mortality than receiving MV in the presence of no maternal antibody. The trend was similar for two different strains of MV. No child who had received MV died of measles infection and the differential effect of MV in the presence of pre-vaccination measles antibody is therefore non-specific.

### Strengths and Limitations

It could be speculated that those with baseline measles antibodies were healthier than those with undetectable levels and therefore had better survival. However, as in previous studies (13), having low detectable levels of antibody was not associated with a benefit in the children who did not get an active MV at enrolment.

There is no way of knowing exactly whether the children with detectable pre-vaccination antibody had maternal antibody or antibody due to measles exposure (18, 19). However, maternal antibody is waning during infancy and would therefore probably be in the lower end of the detectable scale among children who are 4–9 months old, whereas children who have had natural measles infection are likely to have high levels (Table 1) (15). We therefore tested those with low levels against those with undetectable levels.

We previously showed that children who had measles infection in the first 6 months have increased mortality throughout childhood (18, 19). Hence, children with higher antibody levels following earlier exposure might be at increased risk of death. The inclusion of such children in the analyses could confound the analysis of the impact of MV in the presence of “maternal” antibody. However, when we compared any level of antibody with undetectable levels, the beneficial effect of having antibodies was essentially the same. Hence, it would matter little where we set the cut-off for low detectable antibody levels. Also, studies from Haiti and Bangladesh suggest that MV to children with a history of measles infection may also be beneficial for child survival (20–22). Hence, it is not inconsistent that those children with pre-vaccination antibodies over 250 mIU/ml had similarly low mortality as the children with low measles antibody levels.

### Consistency With Previous Observations

The present study is consistent with the previous observation of the benefit of receiving MV early in the presence of maternal antibody and then having a booster dose of MV around 9 months of age (13). Even if only one dose of MV was given, it was a benefit to have low detectable levels of measles antibody at the time of vaccination. These studies are also consistent with all the epidemiological studies indicating that early MV is better for child survival than later MV (1–7).

The effect on child survival in these studies from the 1980's may be slightly lower than those of early MV from the 1990s and 2000s where the reduction in mortality was 3-fold. The difference may be due to most of the children in the present studies receiving DTP and/or IPV after MV since inactivated vaccines reduce the beneficial NSEs of MV (13, 23, 24). Furthermore, there may have been more natural boosting from wild measles virus during the conduct of these early studies which could have neutralized the difference between group with and without detectable antibody at time of MV.

### Interpretation and Implications

These two studies (6, 13) were conducted in the mid-1980s when most mothers had not been vaccinated and maternal antibodies were therefore mostly due to natural measles infection. There is no indication that the beneficial effect of being vaccinated in presence of maternal antibodies is disappearing over time, since the effect was equally strong in the studies conducted in the 1990s and 2000s, when most mothers would have been measles vaccinated (13). Hence, the beneficial effect of MV in presence of antibody is presumably obtained irrespective of whether maternal antibodies are generated by natural measles infection or vaccination. As maternal antibody levels are declining with improved measles control and less risk of exposure to wild measles virus, most children are likely to have undetectable maternal measles antibody levels when measles vaccinated; hence, it would be interesting to examine whether similar beneficial effect of MV can be obtained in case measles antibody are obtained externally from immunoglobulins.

We have also examined whether maternal priming affected children's response to BCG-vaccination. Two studies have now indicated that it is particularly beneficial to get BCG vaccine if the mother was also BCG-vaccinated (25, 26). An immunological study from Uganda showed stronger pro-inflammatory responses after BCG vaccination of neonates if the mother had a BCG-scar (27). Hence, maternal priming may play a major beneficial role for the child's survival benefit after BCG or MV. Since similar beneficial NSEs have now been found for both MV and BCG vaccinations, the immunological mechanisms explaining the priming effect are likely to be important and should be further studied. These mechanisms have not been examined so far though differential activation of the child's immune system in the presence of maternal antibodies is clearly possible. Transgenerational transmission of epigenetic traits related to host defense has been reported for invertebrates but has not been examine in humans (28). Since maternal priming reduces child mortality with at least 50%, it has to reprogram the immune system in fundamental ways which have been overlooked so far.

It probably makes evolutionary sense that there is a survival advantage to be exposed to infections in presence of maternal immunity which attenuates infections and broadens the immune response (29). This would imply that we should measles vaccinate early when more children still have maternal antibodies, and give a second dose at 9 months of age (3, 4). This could reduce all-cause child mortality substantially in low-income countries with high mortality. We have used this strategy successfully in Guinea-Bissau when two doses of MV at 4.5 and 9 months were associated with a 30% reduction in mortality between 4.5 and 36 months of age (3, 30). In two subsequent RCTs of two-dose MV in Guinea-Bissau and Burkina Faso we found no beneficial effect of early MV (31). During these latter RCTs many mothers were vaccinated and levels of maternal measles antibody were low and consequently fewer children were vaccinated in the presence of maternal antibody. In addition there were numerous campaigns with oral polio vaccine (OPV) and this may have reduced or removed the benefit from early MV (30, 31); we have previously shown that neonatal vitamin A supplementation (NVAS) may also remove the benefit of early MV (3). The marked benefit of early MV may therefore be affected by other interventions. NVAS has not become global policy (32) and OPV campaigns have been stopped or will be stopped soon since OPV is to be replaced by IPV not later than 2024. When used in the future, early MV should be closely monitored to assure that there are no other interfering interventions.

The principle of giving MV in the presence of antibodies contradicts fundamental assumptions of the current global MV programme: VE against measles infection and the effect on general child survival increases with increasing age of vaccination due to the waning of maternal antibody (4, 8). Though studies support that later measles vaccination is associated with a higher antibody response and a slightly better protection against measles infection, it has never been documented that this translate into better child survival by increasing the age of MV (4). Measles vaccinated children have milder measles infection with lower case fatality rate (33); so an improvement in seroconversion may have limited impact on overall survival. In fact, all available studies—including both observational studies and several randomized trials—lead to the opposite conclusion: early MV has a stronger beneficial effect on child survival than later MV (1–7). The policy of increasing the age of MV to 12 months once measles infection has come under control (10) may in fact unintendedly have negative ramifications.

In the era of measles eradication, with most mothers being vaccinated and transferring lower levels of maternal measles antibody, we may need to give the first dose of MV earlier rather than later and/or consider vaccinating adolescent girls with MV to boost their antibody levels prior to pregnancy. We are planning a study to immunize women of fertile age and then vaccinate their future offspring at 4–5 months of age. Hopefully other groups will also examine the implications of boosting maternal antibody levels and measles-vaccinate newborns before 9 months of age.

## Data Availability Statement

The datasets generated for this study are available on request to the corresponding author.

## Ethics Statement

The protocol for the measles vaccine trials was approved by the Danish Central Ethical Committee, the Gambia/MRC Scientific and Ethics committees, and the Guinean Ministry of Health's Research Coordination Committee. Written informed consent to participate in this study was provided by the participants' legal guardian/next of kin.

## Author Contributions

HW and PA designed and conducted the first MV trial in Bissau. CB suggested the analysis of impact of MV in the presence of maternal antibody. PA, HW, CM, AF, and CB are part or the consortium to study the effects of early MV. AA was responsible for the statistical analyses. The first draft was written by CB and PA. CB and PA will act as guarantors of the study. All authors contributed to the final version of the paper.

### Conflict of Interest

During the study period, PA held a research professorship grant from the Novo Nordisk Foundation. The remaining authors declare that the research was conducted in the absence of any commercial or financial relationships that could be construed as a potential conflict of interest.
